# de Winter syndrome or inferior STEMI?

**DOI:** 10.1186/s12872-021-02441-4

**Published:** 2021-12-28

**Authors:** Shijun Wang, Liang Shen

**Affiliations:** Department of Cardiology, Affiliated Hospital of Jiaxing University, No.1882 Zhonghuan South Road, Jiaxing, 314000 Zhejiang People’s Republic of China

**Keywords:** Case report, De Winter syndrome, STEMI

## Abstract

**Background:**

The de Winter electrocardiography (ECG) pattern is associated with acute total or subtotal occlusion of the left anterior descending coronary artery (LAD) characterized by upsloping ST-segment depression at the J point in leads V_1_–V_6_ without ST-segment elevation.

**Case presentation:**

We report an atypical style case of the de Winter ECG pattern accompanied by ST elevation in inferior leads. The patient underwent emergency coronary angiography, which revealed total occlusion of the proximal LAD with no observable stenosis in the right coronary artery.

**Conclusion:**

ECG-related changes in acute total LAD occlusion can present with the de Winter pattern and ST elevation in inferior leads. Recognizing this atypical ECG pattern is critical for immediate reperfusion therapy.

## Background

A de Winter ST-T-related change can present with acute subtotal or total left anterior descending (LAD) occlusion. The de Winter electrocardiography (ECG) pattern is denoted primarily by upsloping ST-segment depression at the J point in leads V_1_–V_6_ without ST-segment elevation. Here we report the case of a patient with acute total occlusion of LAD who presented with an ST elevation in inferior leads, up-sloping ST-segment depression in V_2_ to V_5_ leads, and specular alterations in the ST segment in leads I and aVL.

## Case presentation

**Fig. 1:**
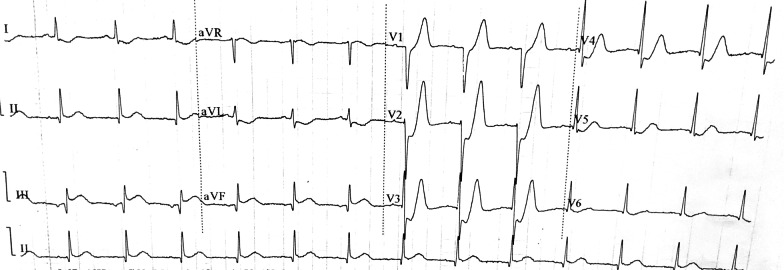
ECG obtained in the emergency room

Our patient was a smoker in his 40 s with a medical history of hypertension. He presented to the emergency department with persistent chest pain for 2 h. Once admitted to emergency room, a 12-lead ECG (Fig. 1) was performed and the result showed ST-segment depression in V_2_ to V_5_ leads, followed by positive and symmetrical T waves, alongside II, III, and aVF lead ST elevation and I, aVL lead ST depression. Meanwhile, troponin I level was tested and the result was 0.17 ng/mL (reference range, 0–0.11 ng/mL).

**Fig. 2:**
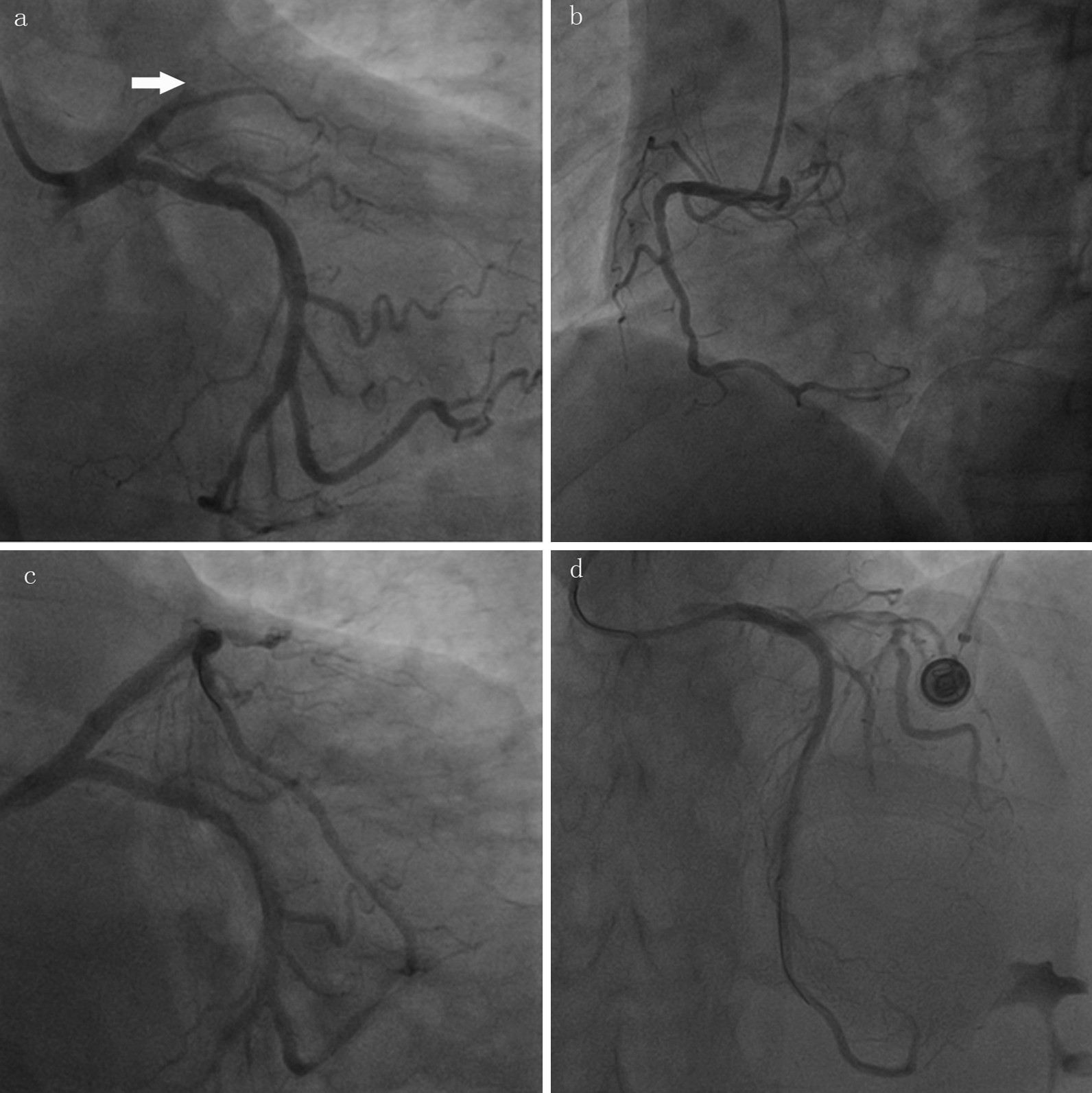
**a**, **b**, Coronary angiography revelation of total occlusion of the proximal LAD (white arrowhead) and slight stenosis in the right and left circumflex coronary arteries; **c**, **d** Coronary angiography after stent implantation

**Fig. 3:**
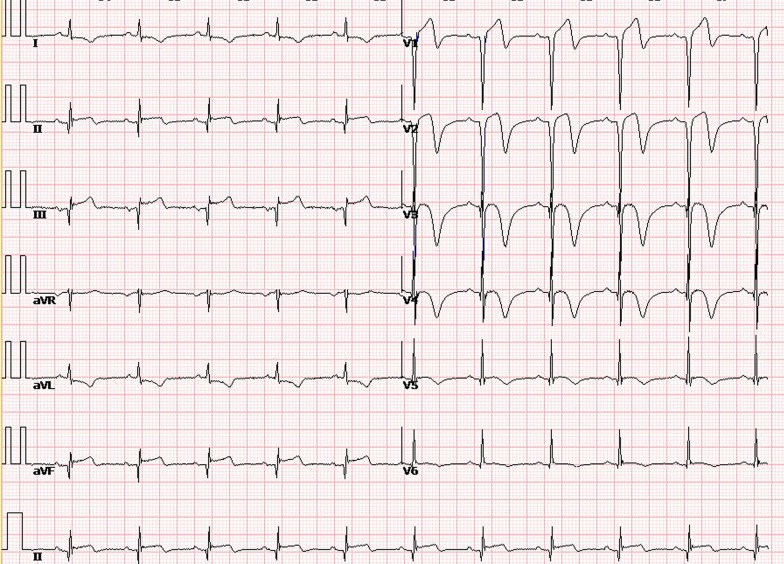
ECG recording 12 h after revascularization

**Fig. 4:**
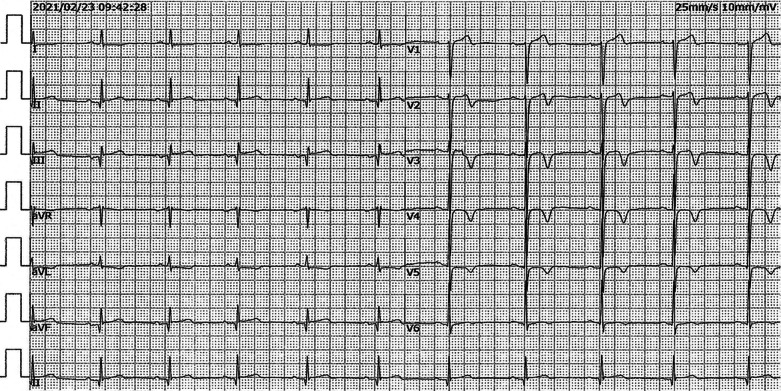
ECG recording 1 month after PCI

Based on the ECG style, acute subtotal occlusion of LAD was subsequently suspected, and urgent coronary angiography was performed, which revealed total occlusion of the proximal left anterior descending artery (LAD) and slight stenosis in the right and left circumflex coronary arteries (Fig. 2a, b). As a result, primary percutaneous coronary intervention (PCI) was carried out, and a drug-eluting stent was implanted successfully in the LAD artery (Fig. 2c, d). The patient was completely relieved of his symptom after revascularization. Another ECG (Fig. 3) recorded 12 h after reperfusion showed elevations in Q waves in leads II, III, and aVF. It also showed that the T wave reverted to inversion in leads V_2-6_, as did q waves in leads V_2-4_, consistent with the ECG evolution of acute myocardial infarction. Six days later, the patient received cardiac rehabilitation counseling and was discharged. And one month after discharge, a follow-up ECG recording revealed that the ST-segment in leads II, III, and aVF was restored to baseline, and q waves disappeared in leads V_2–5_ and were replaced by r waves (Fig. 4).

## Discussion

In 2008, de Winter et al. [1] first described a new ECG sign of proximal LAD occlusion characterized mainly by up-sloping ST-segment depression at the J point in V_1–6_ leads, followed by tall and symmetrical T waves that accounted for approximately 2% of patients with subtotal or total occlusion of the proximal LAD artery. Then in 2018, Tsutsumi et al. [2] reported the de Winter sign in inferior leads. In our case, we illustrated an atypical style of the de Winter ECG pattern accompanied by ST elevation in inferior leads alongside specular alterations in lateral leads. To the best of our knowledge, this is the first case to present with the de Winter pattern alongside the inferior STEMI.

The mechanisms of the de Winter ECG pattern are still to be elucidated fully. Yet, it is believed that ST-segment elevation does not occur without the activation of sarcolemmal adenosine triphosphate (ATP)-sensitive potassium channels [1]. A difference in sensitivity to ischemia between endocardium and epicardium [3] and collateral blood supply might also contribute to this special ECG pattern.

Specularity is not a typical sign of de Winter pattern, since ischemia has not been transmural yet. However, we observed specular alterations of the ST segment in lateral leads, which might due to the different sensitivity to ischemia between inferior and anterior wall. ST depression in lateral leads is supposed to be the result of transmural ischemia in inferior wall.

Reportedly, the de Winter ECG pattern is transient and dynamic [4, 5]. Patients with this ECG pattern can evolve to present with ST-segment elevation in precordial leads and vice versa, possibly due to thrombus formation and autolysis [6]. In the present case, although LAD was totally occluded, we did not observe a dynamic ECG pattern, perhaps because we failed to detect pre-procedural ECG.

## Conclusion

The inferior lead ST elevation in our case might have stemmed from a dominant left coronary artery and posterior descending artery derived largely from LAD. The LAD bypasses the apex of the heart and supplies part of the inferior myocardium. The posterior descending artery derived from LAD also provides blood supply to the septal and inferior myocardium. Once LAD is occluded, the blood flow supplied to the inferior myocardium is interrupted, leading to inferior myocardial infarction. Yet, emergency reperfusion therapy is recommended regardless of the diagnosis type: de Winter or inferior STEMI.

## Data Availability

All available information is contained within the present manuscript.
